# Correlations between Persistent Olfactory and Semantic Memory Disorders after SARS-CoV-2 Infection

**DOI:** 10.3390/brainsci12060714

**Published:** 2022-05-31

**Authors:** Julie Fiorentino, Magali Payne, Elisa Cancian, Alexandra Plonka, Louise-Émilie Dumas, David Chirio, Élisa Demonchy, Karine Risso, Florence Askenazy-Gittard, Nicolas Guevara, Laurent Castillo, Philippe Robert, Valeria Manera, Clair Vandersteen, Auriane Gros

**Affiliations:** 1 Département d’Orthophonie de Nice, Faculté de Médecine de Nice, 06107 Nice, France; magalipayne04@gmail.com (M.P.); alexandra.plonka@outlook.com (A.P.); phil.robert15@orange.fr (P.R.); valeria.manera@univ-cotedazur.fr (V.M.); auriane.gros@univ-cotedazur.fr (A.G.); 2 Laboratoire CoBTeK, Institut Claude Pompidou, Université Côte d’Azur, 06100 Nice, France; dumas.le@pediatrie-chulenval-nice.fr (L.-É.D.); askenazy.f@pediatrie-chulenval-nice.fr (F.A.-G.); vandersteen.c@chu-nice.fr (C.V.); 3 Institut Universitaire de la Face et du Cou, Centre Hospitalier Universitaire, Université Côte d’Azur, 06100 Nice, France; cancian.e@chu-nice.fr; 4 Institut NeuroMod, Université Côté d’Azur, 06902 Sophia-Antipolis, France; guevara.n@chu-nice.fr (N.G.); castillo.l@chu-nice.fr (L.C.); 5 Hôpitaux Pédiatriques de Nice CHU-LENVAL, 57 Avenue de la Californie, Centre Hospitalier Universitaire, Université Côte d’Azur, 06200 Nice, France; 6 Département de Médecine Infectiologique, Hôpital de l’Archet, 151 Route de Saint-Antoine, Centre Hospitalier Universitaire, Université Côte d’Azur, 06200 Nice, France; chirio.d@chu-nice.fr (D.C.); demonchy.e@chu-nice.fr (É.D.); risso.k@chu-nice.fr (K.R.)

**Keywords:** COVID-19, cognitive disorders, olfactory dysfunction, olfactory testing, semantic memory

## Abstract

**Background**: One of the main symptoms of COVID-19 is hyposmia or even anosmia. Olfactory identification is most often affected. In addition, some cognitive disorders tend to appear following the infection, particularly regarding executive functions, attention, and memory. Olfaction, and especially olfactory identification, is related to semantic memory which manages general knowledge about the world. The main objective of this study was to determine whether semantic memory is impaired in case of persistent post COVID-19 olfactory disorders. **Methods**: 84 patients (average age of 42.8 ± 13.6 years) with post COVID-19 olfactory loss were included after consulting to the ENT department. The clinical evaluation was carried out with the Pyramid and Palm Tree Test, the word-retrieval task from the Grémots, the Sniffin’ Sticks Test and the Computerised Olfactory Test for the Diagnosis of Alzheimer’s Disease. **Results**: Semantic memory was impaired in 20% (*n* = 17) of patients, especially in the 19–39 age-group. The olfactory threshold was only significantly correlated with the semantic memory scores. **Conclusions**: Similar to all cognitive disorders, semantic disorders can have a negative impact on quality of life if left untreated. It is essential to carry out specific assessments of post COVID-19 patients to accurately determine their disorders and to put in place the best possible rehabilitation, such as speech and language therapy, to avoid quality-of-life impairment.

## 1. Introduction

SARS-CoV-2 infection causes many symptoms, including olfactory and gustatory dysfunctions [1,2,3], mostly in women [1,4,5,6]. Acute olfactory disorders prevalence varies between 5.1% and 98% according to studies [4,5,7] but resolve spontaneously, totally, or partially, within 8 weeks for 83.2% of patients [8]. After 6 months post-infection, recovery is almost complete for 95% of patients [9]. Despite spontaneous recovery, 36.5% of patients still complain of persistent olfactory disorder after 6 months post-infection [10].

Olfaction is mediated by a complex olfactory system based on a chemo-electric transduction [11,12]. The olfaction signal begins with an odorant compound binding an odorant binding protein on a population of olfactory neurons within the olfactory epithelium overlaying the medial aspects of superior turbinate and (posterior) middle turbinate and the facing septal surface. An electric signal then progresses through the 6 layers of olfactory bulb to mitral cells (2nd neuron) and afterwards (3rd neuron) to the primary olfactory cortex (piriform cortex, anterior olfactive nucleus, olfactive tubercule, anterior amygdaloid complex, entorhinal and parahippocampic cortex). Beyond that, neuronal connectivity extends to secondary cortical areas, specifically orbito-frontal and hippocampic cortex. In COVID-19, the viral membrane glycoprotein S1, facilitated by TMPRSS_2_ [13], binds with ACE2 which is widely expressed on olfactory epithelium non-neuronal cells. Due to deep damages of that infected neuroepithelium, more and more reports suggest the penetration of SARS-CoV-2 into the central nervous system [14,15] through the olfactory cleft and more particularly through sustentacular and/or trans-cribriform sheath cells [13,16,17].

Olfactory loss is a consequence of many viral upper respiratory tract infections such as infuenza virus, rhinoviruses or parainfluenza viruses. However, olfactory disorders seemed to be more recurrent and severe following SARS-CoV-2 infection [18]. In addition, in view of these olfactory disorders persitence as well as the presence of cognitive disorders in some patients following COVID-19, the hypothesis of an invasion of the central nervous system was raised by several authors [19].

Indeed, studies concerning COVID-19 tend to show an impaired cerebral functioning especially in regions involved in olfaction and cognitive abilities, such as the frontal cortex, the hippocampus, the amygdala, the cerebellum, and the insula [20,21,22,23,24,25]. A recent study using pre- and post-infection imaging data identified a reduction in grey matter thickness, especially in the left hemisphere, and tissue-contrast in the orbitofrontal cortex and parahippocampal gyrus, which are highly connected to the olfactory system [26]. The damage to certain brain regions as a result of SARS-CoV-2 infection seems to lead to cognitive dysfunction [20,24,25,26,27,28,29,30]. Indeed, the percentage of post COVID-19 patients with global cognitive impairment varies from 15 to 80% [31]. This cognitive dysfunction results in executive disorders [20,25,26,27,28,29,30,31,32,33], attentional disorders [20,26,28,30,32,33], memory disorders [24,27,28,30,32,33], and sometimes language disorders regarding semantic and phonological verbal fluency [25,30,32,33].

Regarding olfaction, long-lasting olfactory disorders seem to impair odor identification (OI) more than odor detection threshold (OT) and discrimination (OD) [34]. The neurological processing of olfaction involves cortical and subcortical areas and some specific structures also used in language and memory processing, such as the frontal and temporal cortex [35,36,37], the amygdala-hippocampal complex [38] and the insula [39]. In addition, it has been shown that olfactory identification is influenced by executive functions and semantic memory [40,41], as well as verbal episodic memory [42]. Semantic memory is a long-term memory containing general knowledge about the world such as concepts, word meanings, knowledge about objects, places, or people. It can be divided in two parts: semantic representations processing, which can be evaluated with verbal fluency and generative naming, and semantic representations themselves, which can be evaluated with matching task [43,44]. A study with brain injured people also revealed a correlation between olfactory identification skills and semantic memory skills [45]. Consequently, olfaction, language and memory are correlated [46]. Moreover, the greater impairment of OI compared to OT suggests an involvement of olfactory brain structures [47,48].

Therefore, patients suffering from long lasting post COVID-19 olfactory disorders seem to have central, cognitive and identification olfaction subdimension impairments.

Thus, the objective of this study is to investigate whether there is an impairment of semantic memory in post COVID-19 patients. If so, it seems relevant to investigate whether persistent olfactory disorders and semantic disorders are correlated. These findings would permit the identification of patients at risk of semantic disorders. In addition, it could allow patients to receive appropriate health care, adapted to their disorders and complaints. It would also deepen our knowledge about COVID-19 neurological consequences.

## 2. Materials and Methods

### 2.1. Study Design and Population

This monocentric study was approved by the institutional review board of the Nice University Hospital (CNIL number: 412). It is part of a large work registered under a ClinicalTrials.gov number (ID: NCT04799977). Patients had post COVID-19 persistent olfactory disorders which had motivated a consultation at the ENT department of the local University Hospital where they were recruited from November 2020 to January 2022.

Patients were mainly self-referred or referred by general practitioners or ENT colleagues. They had to be over 18 years of age, had a proven COVID-19 infection and complaining from an olfactory loss for more than 6 weeks after infection. Patients with a personal history of olfaction disorder, ENT cancer, head radiotherapy history, neurodegenerative disease, post viral (before the pandemic) olfactive history or language-related pathologies, were not included. Almost all patients report an RT-PCR-proven SARS-CoV-2 diagnosis. When they did not have an RT-PCR but only a chest-CT highly suggesting COVID infection (performed when febrile pneumonias were diagnosed in pandemic conditions), SARS-CoV-2 infections were always secondarily confirmed by serology (IgG positivity). We retrospectively extracted patients’ demographic data and clinical features including medical history and nasofibroscopies results.

### 2.2. Semantic Assessment

Semantic memory was assessed with the word-to-word matching task from the *Pyramids and Palm Trees Test* (PPTT) [49,50]. It consists of 52 triads of written words presented on a computer. The stimulus was placed in the middle of the top of the screen, while the target (the semantically closest word) and the distractor were placed on either side of the bottom of the screen, below the stimulus. The subject had to identify the target and click on it. The analysis of the score obtained considers the age and socio-cultural level of the subject through previously published normative values [50]. The 5% cut-off score corresponds to a Z-score of −1.65. Thus, a low score indicates an important semantic impairment.

### 2.3. Word Retrieval

A generative naming test was used to determine whether semantic memory itself was impacted or whether it was access to the lexical store [51]. The generative naming test used belongs to *Grémots battery: Evaluation du langage dans les pathologies neurodégénératives* [52]. It is composed of 36 colored pictures that can be divided in two lexical categories: biological and manufactured. Nouns were selected according to three linguistic criteria: frequency, syllabic length, and lexical category. Pictures were presented to the subject one after the other, and the subject was asked to name them. Subject’s correct answers provided a strict and a broad score out of 36. Strict score corresponded to correct answers produced within 5 s. Broad score was calculated by the sum of correct answers produced with an arthritic disorder or phonemic paraphasias within 5 s, and correct answers produced after a delay (between 5 and 10 s) or after self-correction. Scores analysis takes the subject age as well as his socio-cultural level into account.

### 2.4. Olfactory Assessment

Olfactory function was assessed using two tests: *Sniffin’ Sticks Test* (SST) [53,54] and *Test Olfactif informatisé pour le Diagnostic de la maladie d’Alzheimer et de l’Apathie* (TODA patent application filed on 28 September 2021) [55].

The SST is a psychophysical test validated in several European countries. It has three subtests: odor Threshold detection (T), odor Discrimination (D) and odor Identification (I) [53]. Odorants are presented in pens tips. For the first two subtests, subjects are blindfolded. The T test was measured by a forced choice task among 16 triplets of pens. Among every triplet, one felt pen tip was impregnated with phenylethyl alcohol (PEA) diluted in an increasing concentration of solvent. The two other felt pens were impregnated with a non-odorous solvent. The three pens were presented to the subject in a random order, with the instruction (forced choice) to find the PEA pen. The D test also included 16 triplets of felt pens. Within a triplet, two pens were impregnated with the same odorant while the third was impregnated with a different odorant. By forced choice, the subject had the instruction to identify the pen with a different smell. The I test consisted in presenting the 16 felts one after the other. The subject’s task was to choose from a list of 4 written proposals, the one corresponding to the identified odor. The sum of these three subtests correct answer scores gives a global olfactory score called « TDI ». A TDI score ≤ 16 indicates functional anosmia, a score between 16.25 and 30.5 indicates hyposmia and a score > 30.75 indicates normosmia [54].

Olfaction evaluation was completed with the TODA test, developed by Côte d’Azur University and Nice University Hospital, and included a 14 fragrances kit and an application recording the test and results [55]. These 14 odorants were in compact jars filled with paraffin wax. They were divided in two categories: biological (citrus, chocolate, strawberry, mint, coconut, rose, vanilla, almond, jasmine, lavender, and pear) and manufactured (wood, grass and clean). Each odorant was diluted in 4 concentrations and filled in 4 different compact jars: concentration 1 varied between 1 and 5% of olfactory raw material; concentration 2 is 10% dilution, concentration 3 is 20% dilution and concentration 4 is 40% dilution. Six scents were randomly and successively presented to the subject. First, the subject had to confirm an odorant perception with the less concentrated container (concentration 1). In case of lack of perception, higher concentrations were presented until all four were tested. If there was still a lack of perception at the fourth concentration, we presented the next odorant. When the odor was perceived, the subject had to identify it by choosing between 4 illustrations, 3 of them being semantic distractors.

Two scores were determined:

- The odor detection threshold ranges from 1 to 5. This score represented the average intensity at which the subject perceived the odor. A low score indicated a preserved odor detection ability.
- The odor identification score, out of 6. It represented the number of correctly identified fragrances. A high score indicated a preserved odor identification ability.

### 2.5. Statistical Analysis

To investigate correlations between olfactory abilities (TDI and TODA scores) and semantic skills (PPTT and generative naming scores), non-parametric correlations (Spearman rho) were employed as most of the data did not follow a normal distribution (as confirmed by Shapiro-Wilks tests). Results were considered as statistically significant when they met a bilateral alpha level of 0.05. Given the exploratory nature of the present study, we did not correct the alpha level for multiple comparisons.

## 3. Results

### 3.1. Demographic and Clinical Features

In this case, 84 patients were included in this study. As defined by WHO organization, every patient was considered as long COVID-19. Demographics and clinical data are reported in Table 1. Here, 16 patients suffered from well managed chronic rhinosinusitis (CRS) including only CRS without polyps. Among patients included, 88.1% (*n* = 74), 4.8% (*n* = 4) and 7.1% (*n* = 6) had, respectively, a mild form, a moderate form with conventional hospitalization and finally a severe form of the disease with hospitalization in an intensive care unit. All nasofibroscopies were normal. Demographic and clinical characteristics are described in Table 1.

Since COVID-19 infection, 20% (*n* = 17) reported fatigue, 13% (*n* = 11) reported language disorders (such as lack of words), 20% (*n* = 17) reported cognitive disorders (such as attention and concentration difficulties, memory loss) and 26% (*n* = 22) reported psychological distress (such as impaired quality of life, anxiety and depression).

Their educational background varied from no education to more than 13 years of education: 1.2% (*n* = 1) had between 0 and 5 years of education, 32,1% (*n* = 27) had between 5 and 9 years of education, 61,9% (*n* = 52) had over 12 years of education and 4,8% (*n* = 4) didn’t indicate their education level.

We followed the age grouping carried out by Callahan et al. (2010) [50] as we were using their normative data for PPTT scores. However, we decided to group by 20-year age groups rather than by 10-year age groups: the division into 10-year age groups would have created groups with too few patients in some of them, making statistical analyses inappropriate. Subjects were divided into 3 age groups: (A) 19–39 years (*n* = 35); (B) 40–59 years (*n* = 40); and (C) 60 years and over (*n* = 9).

### 3.2. Semantic Abilities

Descriptive analyses about semantic abilities are reported in Table 2. No significant correlations were identified concerning semantic abilities and medical history, or dedicated COVID-19 treatment used during infection.

#### 3.2.1. Semantic Memory

A significant moderate correlation between age and the *Pyramid and Palm Tree Test* was observed (rho(82) = 0.314, *p* = 0.0036). Scores showed that 17 patients (20.2% of total population) had an impaired semantic memory. 16 (94%) were from group A and 1 (6%) was from group B. Of these, according to their TDI scores, 5 (30%) were anosmic, 6 (35%) were hyposmic and 6 (35%) were normosmic. The 3 most failed items were “Eskimo-Kayak” (failed at 60.7%), “Bellows-Fire” (failed at 39.3%) and “Mill-Tulip” (failed at 28.6%).

#### 3.2.2. Word Retrieval Ability

Strict scores, broad scores and time were within the norms indicating a preserved word retrieval ability. We considered the score to be pathological when it was below or equal to the 10th percentile. Due to technical problems, only 53 patients have a strict score, 45 have a broad score and 32 have a time score. However, correlations between PPTT scores and the generative naming time scores were statistically significant and large for the 40–59 age group (rho(16) = −0.533, *p* = 0.0227).

### 3.3. Olfactory Capacities

Results of the SST showed that 21.4% (*n* = 18) of the patients could be classified as normosmic, (TDI ≥ 30.75), 57.1% (*n* = 48) as hyposmic (16.25 ≤ TDI ≤ 30.5) and 21.4% (*n* = 18) as anosmic (TDI ≤ 16). In all subscales, some scores were below the cut-off score: 46 T scores (54.8% patients), 44 D scores (52.4%), 53 I scores (63.1%) and 56 TDI scores (66.7%). Regarding the population, group C had the lowest T, D and TDI means (T-SST = 3.72 ± 2.7, D-SST = 8.56 ± 2.2, TDI = 22.72 ± 4.7) which were in accordance with normative data for this age-group. Group A had the lowest identification mean (I-SST = 9.40 ± 3.9) which was under the percentile 10 for this age-group.

Concerning TODA threshold scores, 46,4% of patients (*n* = 39) had a score below or equal to 1, 47.6% (*n* = 40) had a score between 1 and 3, and 6% (*n* = 5) had a score superior or equal to 3. Identification scores were below 3 for 15 patients (17.9%) and superior or equal to 3 for 69 patients (82.1%). Flower scents were the most misidentified (46.5% was recognized) followed by manufactured scents (50.3% was recognized). Patients made a total of 303 errors of which 109 (36%) were semantic errors. Group A had the worst means for all TODA scores (T-TODA = 1.66 ± 0.97, I-TODA = 3.97 ± 1.8). Concerning all the population, correlations between threshold scores and identification scores were statistically significant and large (rho(82) = −0.5653, *p* < 0.01). No significant correlations were identified concerning olfactory capacities and medical history or dedicated COVID-19 treatment used during infection.

### 3.4. Correlations between Olfactory Disorders and Semantic Memory

**Figure 1:**
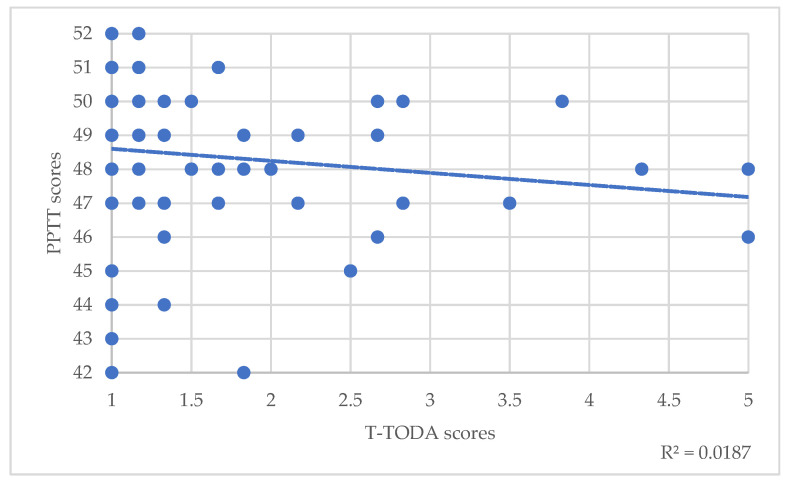
Correlations between PPTT scores and T-TODA scores. The dotted line represents the trend line.

**Figure 2:**
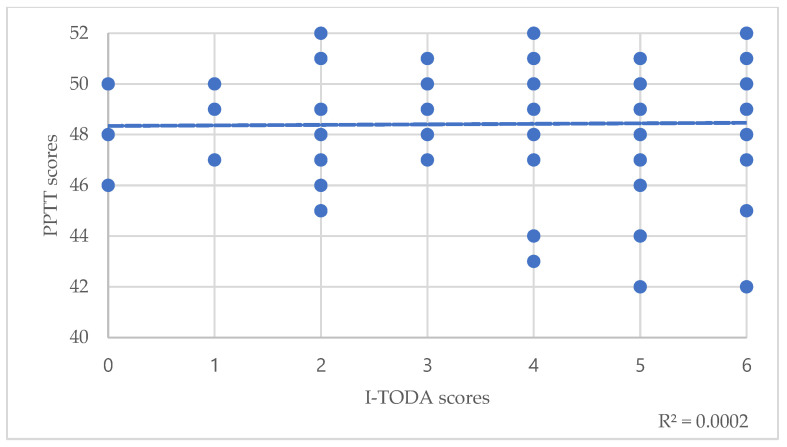
Correlations between PPTT scores and I-TODA scores. The dotted line represents the trend line.

Regarding the total population, Spearman’s correlations between PPTT scores and T scores of the TODA suggested a small significant correlation between semantic memory and odor threshold detection (T-TODA rho(82)= −0.24, *p* = 0.025) (Table 3, Figure 1). Correlations with odor identification weren’t significant (I-TODA, rho(82)= 0.075, *p* = 0.49) (Table 3). However, trend lines showed that the lower these scores were, the lower the PPTT score was (Figure 2).

**Figure 3:**
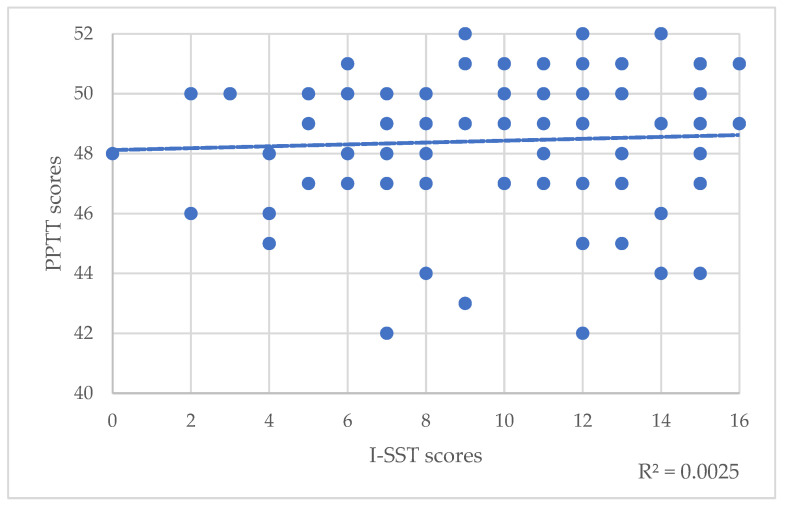
Correlations between PPTT scores and I-SST scores. The dotted line represents the trend line.

Correlations between PPTT scores and the four SST scores were not statistically significant regarding the total population (Table 3). However, the trend line regarding correlation between PPTT scores and I-SST scores revealed that the lower the I-SST score, the lower the PPTT score (Figure 3).

Out of the 17 patients with a PPTT score below the cut-off score, 9 had a T score of the SST below the cut-off score and 3 with a D score below the cut-off score. 2 of them had both T and D scores below the cut-off score.

### 3.5. Correlations between Olfactory Disorders and Word Retrieval

Regarding the total population, Spearman’s correlations between the three generative naming scores and the four SST scores were not statistically significant. In the same way, correlations between the three generative naming scores and the TODA scores were not significant (Table 3). However, correlations between generative naming time score and T scores of the TODA were statistically significant and moderate for the 40–59 age group (rho(16) = 0.48, *p* = 0.04).

## 4. Discussion

### 4.1. Semantic Memory Impairment Following SARS-CoV-2 Infection

Semantic memory of COVID-19 long-haulers patients was evaluated as well as their olfactory functioning. To our knowledge, this study is the first to investigate semantic memory in patients infected with SARS-CoV-2. It appeared that 20% of the population had an impaired written verbal semantic memory, mainly among the 19–39 age group. One of the most failed items was culture-specific [50] though the population’s educational background was sufficiently high not to have expected difficulties with it. This result is consistent with studies showing temporal, hippocampal [56,57] and parahippocampal [25,26] brain damage with a reduction in grey matter particularly in the left hemisphere [26] following SARS-CoV-2 infection. Indeed, semantic memory relies heavily on the temporal lobe as well as the hippocampus and parahippocampal regions [58,59] with a greater involvement of the left hemisphere [60]. Several studies also reported an impaired semantic verbal fluency [30,32,33] in patients infected with SARS-CoV-2. Following the result of our study, we may advance the hypothesis that semantic verbal fluency impairment is due to the impairment of semantic memory as in neurodegenerative diseases, as also suggested by other studies [61].

A review of cognitive disorders in COVID-19 has shown that, although results indicate possible language impairment, few studies used domain-specific language tasks [31]. In addition, tasks used in studies to assess semantic memory are linked and mechanisms of control involve semantic representation processing (as verbal fluency and generative naming) more than semantic representation itself. Using a matching task, we wanted to study more particularly semantic representation which depends less on executive functioning.

Thus, this study also sought to assess objectively word retrieval ability. A previous study indicated that 59.5% of post COVID-19 patients reported tip-of-the-tongue word finding problems [62]. However, in our study, word retrieval ability wasn’t impaired. Therefore, generative naming task score is not correlated with performance in semantic representation task, that confirm two different systems [63].

Though, PPTT scores and generative naming time scores were statistically correlated for the 40–59 age group as will be developed later in the discussion. It suggests that naming time scores can be more of interest to study mild semantic representations deficits such as those shown in studies on AD.

### 4.2. Correlation between Olfactory Disorders and Semantic Memory

Olfactory neuroepithelium is one of rare neuroepithelium to be able to regenerate [12]. Global basal cells, near olfactory epithelium basal membrane, could differentiate into non neuronal and neuronal olfactory cells and could restore olfactory bulb rhinotopy [64], facilitated by the help of unsheathing cells [65]. Regeneration process is corrupted in post COVID-19 persistent olfactory disorders with up to 30% of patients complaining from olfactory loss 1 year after the infection [66] and 40% [67] progressive onset of parosmias parallel to olfactory recovery. Parosmias peripheral origin is supported by an abnormal neuronal regrowth, including bad proximity neurons contacts, probably worsened by corrupted unsheathing cells [13,16,17], in a hypotrophic olfactory bulbs environment [68]. Parosmia central origin is supported by gray matter alterations [69] and olfactory cortex hypometabolism [70]. As COVID-19 targets the neuroepithelium and probably spread into the central nervous system and so olfactory semantic network, these peripheral and central olfactory impairments may contribute to semantic networks dysfunction.

Regarding olfactory functions, it appeared that the 60+ age group had the worst average SST scores except for the I score with the 19–39 age group having the lowest average. This age group also had the worst averages at TODA and PPTT scores. Furthermore, as identified in a previous study [34], SST I score appeared to be worse than T and D scores for all the population, inducing a greater impairment of olfactory identification.

One of the objectives of this study was to determine whether there was a correlation between olfactory disorders and semantic abilities in post COVID-19 patients. There was no correlation between SST (general and sub scores) and PPTT scores however PPTT scores significantly correlated with some TODA scores. This difference in correlation between the two olfactory tests (SST and TODA) and the semantic memory test (PPTT) can be explained by the larger panel of odors used in the TODA test. Indeed, in this test, threshold score is determined by using six different odors with four dilutions for each while only one odor is used in the SST. In addition, the semantic aspect of olfaction was particularly considered when this test was created. Effectively, this test was designed for the early diagnosis of Alzheimer’s disease. This disease particularly affects odor identification at an early stage, whereas threshold and discrimination are preserved in the early stages of the disease, revealing an impairment of the olfactory semantic memory. This is due to alterations in the entorhinal cortex, hippocampus, and orbitofrontal cortex, which are also regions that manage semantic memory [55]. Therefore, this test contains semantically related items highlighting mild semantic impairment more easily. Thus, regarding the entire population, PPTT scores were significantly correlated with TODA threshold scores, but the correlation was of small effect size.

This is in line with results found in previous studies, revealing the association of olfactory and cognitive abilities in post COVID-19 patients [29,71,72]. In addition, it was also suggested that the more COVID-19 symptoms were more severe and persistent, such as olfactory impairment, the more cognitive impairment was higher [29,62,72]. Our results support this observation: the higher were TODA threshold scores, revealing deteriorated olfactory perception, the lower were PPTT scores.

Furthermore, because of the important links between olfactory identification and semantic memory and the greater impairment of olfactory identification in post COVID-19 patients [34], we expected to find a correlation between the SST and/or TODA identification scores and PPTT scores but none was found for the whole population. However, threshold and identification scores of the TODA were significantly correlated revealing that the more the threshold is impaired, the more the identification is impaired. In addition, trend lines revealed that the higher the PPTT score, the higher the identification score of both SST and TODA. Thus, these findings seem to support a central alteration in post COVID-19 patients. It would therefore appear that verbal semantic memory is well impacted following COVID-19, with 20% of the population affected, but with no statistically significant link to olfactory semantic memory.

### 4.3. Correlations between Olfactory Disorders and Other Cognitive Functions

Several studies have also shown an impairment of executive functions [20,25,28,29,30,31,32,33] attention [20,26,28,30,32,33] and memory [24,27,30,32,33] following COVID-19. In our study, we found that for the 40–59 age group, generative naming time was correlated with PPTT scores: the longer the generative naming time, the better the PPTT scores. Cognitive tasks seem to take longer for this age group to complete correctly, even if the generative naming times weren’t pathological. This is consistent with the fact that processing speed starts to decrease from the third decade of life and gradually decreases throughout life [73]. However, we found that for the same age group, generative naming time was also correlated with T-TODA scores: the worse the threshold, the longer the generative naming time. Thus, this tends to suggest either an attentional or processing speed alteration which may be related to the SARS-CoV-2 infection. This is in line with a recent study revealing a tissue-contrast reduction in the orbitofrontal cortex [26] which is one of the brain areas governing attentional abilities. The fact that generative naming time and T-TODA scores were correlated is in accordance with studies [32,72] that showed olfactory disorders are associated with attentional, memory and executive function disorders. This result is also in line with studies [62,72] suggesting that the severity and persistence of COVID-19 neurological symptoms influence cognitive impairment extent.

Results of other studies suggest link between semantic tasks and personality traits in AD, with particularly a higher level of openness related to better performance at similarities and verbal fluency test [74]. In COVID-19 studies personality traits are correlated with anxiety and depression, but not cognitive or olfactory function [75].

Correlations showed in our study between olfactory performances and semantic performances are in the same way suggesting potential differences in the pathophysiology of the different symptoms present in post-COVID syndrome. Nevertheless, it would be of interest to verify in future studies if matching tasks performances are correlated to personality traits such as those shown in other task assessing semantic memory (as verbal fluency and naming task).

Finally, this study highlights a specific central consequence of COVID-19: verbal semantic memory appears to be affected in 20% of patients post COVID-19. Cognitive disorders are known to have a significant impact on quality of life [76]. Thus, it seems important to pursue studies concerning cognitive impairment induced by COVID-19, to define in particular which executive functions are impaired, which type of memory and whether these disorders are likely to worsen over time or whether they can be rehabilitated. The mechanisms underlying these cognitive deficits are still poorly understood, the impairment patterns following SARS-CoV-2 infection are many and varied and still require researchers’ full attention.

The main limitations of this study include the lack of a control group or patients’ data prior SARS-CoV-2 infection and the relatively small cohort of 84 patients who consulted spontaneously at the ENT department of local University Hospital, which creates a risk of a recruitment bias. In addition, the exact number of education years was not precisely collected. Concerning tests, we used the PPTT normative data, which was carried out on Quebec population, as the norms concerning French population do not exist. Furthermore, the generative naming test was taken from a test battery for the diagnosis of language impairment in neurodegenerative diseases in patients from the age of 40. As some of the patients in our study were under 40 years of age, this test may not have been the most suitable or sensitive for assessing word-retrieval ability. Future studies with bigger sample sizes, allowing to correct for multiple comparisons, would be needed to confirm the present preliminary results.

## 5. Conclusions

This study focused on the possible impairment of semantic memory in post-COVID-19 patients. The results of the PPTT determined that semantic memory was impaired in 20% of the patients, especially in the 19–39 age-group which also had the lowest scores in olfactory identification. This semantic impairment is correlated with olfactory disorders, as our TODA results suggested, but could stay unseen using only SST. It is therefore important to carry out specific and multidisciplinary assessments of long-lasting post COVID-19 patients with olfactory disorders to identify non olfactory impairments as semantic memory ones. These cognitive disorders could require specific rehabilitations such as speech and language therapy and might be screened as often as persistent olfactory disorders in order to prevent quality of life impairments.

## Figures and Tables

**Table 1:** Demographic and clinical characteristics. SD means Standard Deviation. CT = computed tomography; PCR = polymerase chain reaction; CRS = chronic rhinosinusitis.

	Mean	SD
**Age** (years)	42.8	13.6
**Months post-COVID_19_**	9	4.6
	n	%
**Sex**		
Female	55	65.5
Male	29	34.5
**COVID_19_ testing**		
Molecular PCR test	68	81
Serology (antibody test)	16	19
**COVID_19_ dedicated treatment**		
Oral corticosteroids	12	14.3
Rhino corticosteroids	9	10.7
Inhaled corticosteroids	2	2.4
Azithromycin alone	9	10.7
Azithromycin + Hydroxychloroquine	5	6
Amoxicillin alone	1	1.2
Amoxicillin + Azithromycin	5	6
Others (vitamins, zinc)	22	26.2
**Medical history**		
Smoking	13	15.5
CRS	16	19
Allergies	11	13.1
Asthma	2	2.4
Neurological history (epilepsy)	2	2.4
Diabetes	3	3.6
Cardiovascular diseases	2	2.4
Immunocompromised	2	2.4

**Table 2:** Subtests scores. SD: standard deviation. SST = Sniffin’ Sticks Test; T = SST threshold subtest; D = SST discrimination subtest; I = SST identification subtest.

	Age (Mean ± SD)	Male/Female (*n* = 84)	Months Post COVID-19 (Mean ± SD)	PPTT (Mean ± SD)	Generative Naming (Mean ± SD)			SST (Mean ± SD)				TODA (Mean ± SD)	
				*PPTT* *Scores*	*Strict Score*	*Broad Score*	*Time*	*T*	*D*	*I*	*TDI*	*Threshold Detection*	*Identification*
19–39	30 ± 6.2	11/24	7.4 ± 3.5	47.31 ± 2.63	34 ± 2	34 ± 1	63.93 ± 17.51	4.76 ± 4.04	9.51 ± 3.84	9.40 ± 3.92	23.68 ± 9.68	1.66 ± 0.97	3.97 ± 1.76
40–59	49.3 ± 5.7	14/26	10.4 ± 4.7	49.20±	34 ± 1	35 ± 1	59.46 ± 16.34	4.25 ± 3.26	9.55 ± 3.86	10.15 ± 3.52	23.95 ± 8.61	1.41 ± 0.87	4.19 ± 1.63
60+	65 ± 5.2	4/5	9.6 ± 6	49.3 ± 1.66	35 ± 0.8	36 ± 0.5	54.4 ± 7.1	3.72 ± 2.69	8.56 ± 2.19	10.44 ± 3.32	22.72 ± 4.67	1.22 ± 0.24	4.11 ± 0.78
Total Population	42.8 ± 13.6	29/55	9 ± 4.6	48 ± 2	34.3 ± 1.5	34.9 ± 1.2	61.9 ± 14.6	4.4 ± 3.5	9.4 ± 3.7	9.9 ± 3.6	23.7 ± 8.9	1.5 ± 0.9	4.1 ± 1.6

**Table 3:** Correlation matrix among variables. T = Threshold, D = Discrimination and I = Identification. * *p* < 0.05.

Total Population	T-SST	D-SST	I-SST	TDI	T-TODA	I-TODA
**PPTT**	0.004	−0.121	0.067	−0.009	−0.244 *	0.076
**Generative naming time**	−0.163	−0.170	−0.188	−0.265	0.175	−0.121
**Generative naming strict score**	0.188	0.058	0.148	0.122	−0.16	−0.051
**Generative naming board score**	−0.055	−0.086	0.087	−0.034	−0.146	−0.040

## Data Availability

The data reported are part of an ongoing registration program. Deidentified participant data are not available for legal and ethical reasons. Anonymized data will be made available for research purposes, upon request.
